# PALB2 or BARD1 loss confers homologous recombination deficiency and PARP inhibitor sensitivity in prostate cancer

**DOI:** 10.1038/s41698-022-00291-7

**Published:** 2022-06-29

**Authors:** Kasia M. Dillon, Raie T. Bekele, Zsofia Sztupinszki, Timothy Hanlon, Shahrzad Rafiei, Zoltan Szallasi, Atish D. Choudhury, Kent W. Mouw

**Affiliations:** 1 Department of Radiation Oncology, Dana-Farber Cancer Institute, Boston, MA USA; 2 Harvard Medical School, Boston, MA USA; 3 Danish Cancer Society Research Center, Copenhagen, Denmark; 4 Computational Health Informatics Program, Boston Children’s Hospital, Boston, MA USA; 5 Second Department of Pathology, SE NAP, Brain Metastasis Research Goup, Semmelweis University, Budapest, Hungary; 6 Department of Medical Oncology, Dana-Farber Cancer Institute, Boston, MA USA; 7 Broad Institute of MIT and Harvard, Cambridge, MA USA; 8 Department of Radiation Oncology, Brigham & Women’s Hospital, Boston, MA USA

**Keywords:** Cancer genetics, Prostate cancer, Tumour biomarkers, Predictive markers

## Abstract

PARP inhibitors were recently approved for treatment of molecularly-defined subsets of metastatic castrate-resistant prostate cancer (mCRPC) patients. Although the PARP inhibitor olaparib was approved for use in patients with a mutation in one of fourteen genes, the mutation frequency of the genes varies widely in mCRPC and the impact of the less commonly altered genes on PARP inhibitor sensitivity is uncertain. We used functional approaches to directly test the impact of PALB2 and BARD1 loss on homologous recombination (HR) function and PARP inhibitor sensitivity in prostate cancer cell lines. PALB2 or BARD1 loss led to decreased HR function as measured by loss of radiation-induced Rad51 foci formation as well as decreased HR capacity in a cell-based reporter assay. PALB2 or BARD1 loss also significantly increased sensitivity to the PARP inhibitors olaparib and rucaparib across a panel of prostate cancer cell lines. These data support PALB2 and BARD1 loss as markers of clinically relevant PARP inhibitor sensitivity and highlight the potential to use functional approaches to complement and extend findings from clinical trials of targeted agents.

## Introduction

Prostate cancer is the second leading cause of cancer death in men in the United States, and metastatic castration-resistant prostate cancer (mCRPC) is the terminal disease state responsible for nearly all prostate cancer deaths^1^. Although therapies targeting androgen receptor (AR) signaling remains the backbone of treatment for mCRPC, numerous additional therapeutic strategies are also being investigated to target specific molecular vulnerabilities of mCRPC^2^.

Normal cells have multiple DNA repair pathways that function to repair endogenous and exogenous sources of DNA damage. DNA damage and repair (DDR) gene alterations occur frequently in tumor cells and can have important therapeutic implications. In prostate cancer, multiple large studies have demonstrated that somatic or germline DDR gene alterations are present in 20–25% of prostate tumors^3–5^. The most frequently altered DDR gene in many prostate cancer cohorts is *BRCA2*, a critical member of the homologous recombination (HR) repair pathway that is required for high fidelity repair of DNA double-strand breaks^6^. Whereas both *BRCA2* and *BRCA1* are commonly altered in breast and ovarian cancer, *BRCA1* alterations are less common than *BRCA2* alterations in prostate cancer. Additional genes with known or posited roles in HR are also altered in a subset of mCRPC cases, including *ATM*, *CDK12*, *CHEK2*, and several others^7^.

Alterations in HR genes are of particular clinical relevance because inhibitors of the DDR enzyme poly(ADP ribose) polymerase (PARP) preferentially kill HR-deficient cancer cells^8,9^. PARP inhibitors were first approved for use in HR-deficient breast and ovarian cancer, and recent clinical trials have also demonstrated a role for PARP inhibitors in mCRPC. The PROfound trial randomized molecularly-selected mCRPC patients to the PARP inhibitor olaparib versus a second-generation AR-directed therapy^10^. Two cohorts of patients were enrolled: patients in cohort A had at least one mutation in *BRCA1*, *BRCA2*, or *ATM*, whereas patients in cohort B had a mutation in at least one of twelve additional genes. Progression-free survival (PFS) was significantly longer with olaparib in cohort A as well as in the combined (A plus B) cohort, and these results led to FDA approval of olaparib for mCRPC patients with a tumor alteration in any of 14 genes included in either cohort A or cohort B. Similarly, the TRITON2 trial investigated the PARP inhibitor rucaparib in mCRPC patients with a tumor alteration in *BRCA1*, *BRCA2*, or at least one of thirteen other putative DDR genes^11,12^. The confirmed PSA response rate among patients with a *BRCA1/2* alteration was 54.8%, leading to FDA approval of rucaparib for *BRCA1/2*-altered mCRPC. Work from our group and others has shown that *BRCA2* loss is sufficient to confer PARP inhibitor sensitivity in prostate cancer preclinical models^13,14^.

Beyond *BRCA2*, *ATM* and *CDK12* were among the most commonly altered genes in the PROfound and TRITON2 studies (92/387 [24%] and 49/193 [25%] patients with an *ATM* alteration in PROfound and TRITON2, respectively; 99/387 [25%] and 15/193 [8%] patients with a *CDK12* alteration in PROfound and TRITON2, respectively). Although neither study was powered to specifically assess the impact of *ATM* or *CDK12* alterations, the PARP inhibitor response rates for patients with an alteration in either gene were low: in the TRITON2 study, the PSA response rates for *ATM*- and *CDK12*-altered cases was 4.1% and 6.7%, respectively; in the PROfound trial, the imaging-based PFS for olaparib vs. control was not statistically different for patients either *ATM* (5.4 vs. 4.7 months) or *CDK12* (5.1 vs. 2.2 months) alterations. These data as well as related preclinical work suggest that an *ATM* or *CDK12* alteration may be insufficient to reliably predict PARP inhibitor sensitivity in mCRPC^13,15^. However, a recent analysis of tumor biopsies from patients treated with olaparib on the TOPARP-B clinical trial found that loss of ATM protein expression by immunohistochemistry (IHC) was associated with longer PFS and overall survival than was observed in cases without ATM loss by IHC^16,17^.

**Fig. 1:**
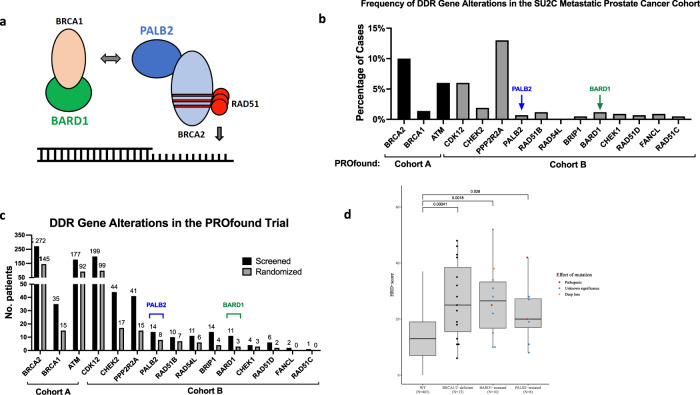
DDR gene alterations and mutational signatures in prostate cancer. **a** Model depicting known interactions among BRCA1, BRCA2, PALB2, and BARD1 at sites of DNA double-strand breaks. **b** Frequency of DDR gene alterations in the SU2C dataset^5^. The genes selected for display are the genes that were included in the PROfound study^10^. **c** Frequency of qualifying DNA repair gene alterations in screened and randomized patients in the PROfound trial of olaparib in metastatic castrate-resistant prostate cancer^10^. **d** HRD scores calculated from WES from TCGA prostate cancer (PRAD) cohort. For each boxplot, the center line, box bounds, and whisker bounds represent the median value, ±interquartile range (IQR), and ±1.5 × IQR, respectively. DDR, DNA damage and repair. SU2C Stand Up to Cancer. TCGA The Cancer Genome Atlas. WES whole exome sequencing.

Alterations in putative DDR genes beyond *BRCA1/2*, *ATM*, and *CDK12* were less common in the PROfound and TRITON2 studies, consistent with data from multiple other large retrospective and prospective cohorts^3–5^. Although alterations in each of these other putative DDR genes are relatively rare (i.e., <1% to ~3%), many of the genes have a well-established role in HR repair in non-prostate tumor contexts. When considered cumulatively, at least 10% of mCRPC cases have an alteration in one or more such genes that have been implicated in HR repair and may therefore sensitize to PARP inhibition. For example, PALB2 (“partner and localizer of BRCA2”) binds directly to BRCA1 and BRCA2 and facilitates BRCA2-mediated Rad51 filament formation to promote HR (Fig. 1a)^18^. Germline mutations in *PALB2* are associated with increased breast and ovarian cancer risk^19,20^ are have also been identified in prostate cancer patients^3,21^. *PALB2*-altered tumors have an HR-deficient mutational signature^22–24^, and data from small numbers of patients suggest that *PALB2*-mutant tumors may respond to PARP inhibition^17,25^. BARD1 (BRCA1-associated RING domain protein 1) forms a heterodimer with BRCA1 and coordinates critical DSB repair events via its E3 ubiquitin ligase activity (Fig. 1a)^26^. Similar to PALB2, mutations in BARD1 can result in cancer predisposition as well as DNA repair defects in tumors^27,28^, raising the possibility that BARD1 alterations may also impact PARP inhibitor sensitivity.

Taken together, available data from non-prostate cancer contexts suggest that alterations in genes such as *PALB2* or *BARD1* can impact HR function and modulate PARP inhibitor sensitivity; however, each of these genes is altered too infrequently in mCRPC to assess the gene-level impact on PARP inhibitor sensitivity in a prospective clinical trial. Therefore, alternative approaches are needed to define the role of each of these genes in mCRPC. Here we use a combination of functional approaches to demonstrate that loss of PALB2 or BARD1 is sufficient to confer an HR-deficient phenotype and drive PARP inhibitor sensitivity in prostate cancer models. These data provide support for PALB2 and BARD1 loss-of-function alterations as biomarkers of sensitivity to PARP inhibition in mCRPC and highlight the importance of using functional studies to complement and extend data from clinical trials.

## Results

### PALB2 and BARD1 alterations and mutational signatures in advanced prostate cancer

We first assessed the frequency of *PALB2* and *BARD1* alterations in advanced prostate cancer. We restricted our analysis to alterations that were likely to lead to loss of function, and therefore considered only nonsense and frameshift mutations as well as deep (homozygous) deletions. The SU2C cohort is comprised of metastatic prostate cancer cases and 3 of 429 (0.7%) cases had a presumed deleterious *PALB2* alteration while 5 of 429 (1.2%) cases had a predicted deleterious *BARD1* alteration (Fig. 1b)^5^. *PALB2* alterations were observed in 14 (1.8%) and 8 (2.1%) of screened and randomized PROfound patients, respectively, whereas BARD1 mutations were present in 11 (1.4%) and 3 (0.8%) screened and randomized patients, respectively (Fig. 1c). In the TRITON2 trial, 2 of 193 enrolled patients (1%) had a *PALB2* mutation and no patients had a BARD1 mutation.

We next examined mutational signatures of HR deficiency in *PALB2-* and *BARD1*-mutant prostate tumors. The HRD score is the sum of three genomic scar-based HR-deficiency measures and can be calculated from next generation sequencing data to infer tumor HR deficiency^29–31^. We calculated HRD scores from 498 publicly available whole exome sequencing datasets from TCGA prostate cancer (PRAD) cohort (Fig. 1d). Tumors with pathogenic germline or somatic *BRCA1* or *BRCA2* biallelic alterations (*n* = 15) had significantly higher median HRD scores than “wild-type” (WT) tumors lacking a *BRCA1/2*, *PALB2*, or *BARD1* alteration (*n* = 466; *p* = 0.00042). Because the number of cases with pathogenic germline or somatic *BARD1* (*n* = 1) or *PALB2* (*n* = 3) were low, we also included mono-allelic events and mutations of unknown significance in our analysis (Supplementary Table 1). We observed that both *BARD1*- and *PALB2*-mutant cases had significantly higher median HRD scores than WT tumors (*p* = 0.023 and *p* = 0.041, respectively), consistent with alterations in *PALB2* or *BARD1* conferring tumor HR deficiency.

### PALB2 or BARD1 loss confers homologous recombination (HR) defect in prostate cancer cells

**Fig. 2:**
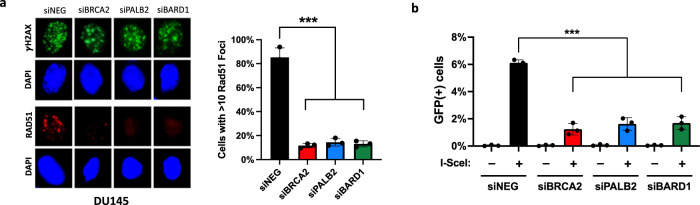
PALB2 or BARD1 loss confers homologous recombination (HR) deficiency in prostate cancer cells. **a** Immunofluorescence analysis showing decrease in radiation-induced RAD51 foci formation in PALB2- and BARD1-depleted prostate cancer cells. All cells form γH2AX foci following radiation, but cells with PALB2 or BARD1 depletion have significantly decreased number of Rad51 foci compared to mock-depleted cells (siNEG). BRCA2-depleted cells are included as a control for HR deficiency. **b** Homologous recombination efficiency as measured by the DR-GFP reporter assay. HR-mediated repair of an induced double-strand break is significantly lower in PALB2- and BARD1-depleted cells compared to mock-depleted cells (siNEG). BRCA2-depleted cells are included as a control for HR deficiency. See Supplementary Fig. 4 for FACS gating strategy. IR ionizing radiation. GFP green fluorescent protein. HR homologous recombination. Error bars represent the standard deviation of three independent experiments. ***p < 0.001.

Given the relatively low frequency of *PALB2* or *BARD1* alterations in prostate tumors, completed and on-going clinical trials are unlikely to enroll a sufficient number of *PALB2*- or *BARD1*-altered cases to provide the statistical power to determine the impact of alterations on response to PARP inhibitors or other agents. Therefore, to gain additional insights regarding the role of *PALB2* and *BARD1* alterations on prostate tumor biology and therapy response, we modeled *PALB2* and *BARD1* loss in the prostate cancer cell lines DU145, LNCaP, and 22Rv1 (Supplementary Fig. 1). We first measured the impact of *PALB2* or *BARD1* loss on HR function. Following radiation, all cells formed phospho-H2AX (γH2AX) foci indicative of DNA damage (Fig. 2a; Supplementary Fig. 2). However, while control (siNEG) cells form Rad51 foci indicative of HR-mediated repair, cells with siRNA-mediated depletion of PALB2 or BARD1 resemble BRCA2-deficient cells and fail to form Rad51 foci, suggesting that PALB2 or BARD1 loss is sufficient to confer HR deficiency in prostate cancer cells (Fig. 2a; Supplementary Fig. 3). Similarly, depletion of PALB2 or BARD1 was also sufficient to confer HR deficiency as measured by the DR-GFP reporter assay that relies on cellular HR machinery to repair an induced DNA double-strand break within a GFP transgene^32^. Whereas control cells were able to restore GFP expression, indicative of intact HR, cells with PALB2 or BARD1 loss had significantly lower levels of HR-mediated GFP expression, similar to the extent observed with BRCA2 loss (Fig. 2b; Supplementary Fig. 4). Taken together, these data indicate that PALB2 or BARD1 loss in prostate cancer cells is sufficient to induce an HR-deficient phenotype.

### PALB2 or BARD1 loss increases sensitivity to PARP inhibition

**Fig. 3:**
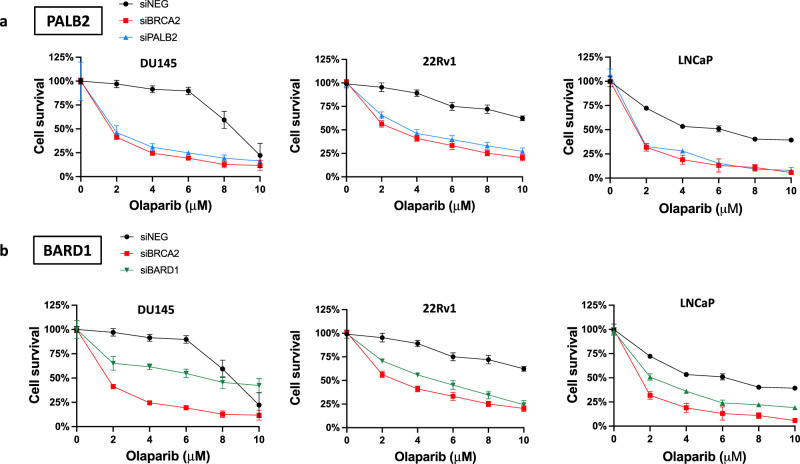
PALB2 or BARD1 loss increases sensitivity to PARP inhibition in prostate cancer cells. **a** PALB2 depletion increases sensitivity to the PARP inhibitor olaparib across prostate cancer cell lines. **b** BARD1 depletion also increases olaparib sensitivity in prostate cancer cell lines. Data points and error bars represent the mean and standard deviation, respectively, of data collected from three experiments.

PARP inhibitors are approved for treatment of HR-deficient tumors in several clinical settings, including recent approvals of olaparib and rucaparib in molecularly-selected mCRPC. Whereas the activity of PARP inhibitors in BRCA2-deficient mCRPC was observed in both the PROfound and TRITON2 trials, alterations in putative HR genes beyond BRCA2 were far less common and therefore the impact of alterations in these genes on PARP inhibitor sensitivity is uncertain. We tested the impact of PALB2 or BARD1 loss on olaparib and rucaparib sensitivity across prostate cancer cell lines and observed a significant increase in sensitivity in PALB2- or BARD1-depleted cells compared to control cells across the cell line models (Fig. 3; Supplementary Figs. 5–8). The impact of PALB2 loss on PARP inhibitor sensitivity was similar to the impact of BRCA2 loss across the cell line models whereas the impact of BARD1 loss on PARP inhibitor sensitivity was less profound than the impact of BRCA2 loss. These data demonstrate that PALB2 or BARD1 loss is sufficient to increase sensitivity to PARP inhibition across prostate cancer cell line models and suggests that PALB2 or BARD1 loss in prostate tumors may be associated with clinically relevant PARP inhibitor sensitivity.

## Discussion

Novel therapeutic approaches are needed to improve clinical outcomes for mCRPC. The recent FDA approval of two PARP inhibitors provides an important treatment option for a subset of mCRPC patients, but challenges remain in identifying the patients most likely to benefit. Olaparib was approved for use in mCRPC patients with a tumor alteration in at least one of fourteen genes based on results from the PROfound study^10^. However, the alteration frequency of each of these genes varied markedly in the trial as well as in other mCRPC cohorts^4,5^. For example, in the PROfound trial, 145 patients with a tumor *BRCA2* alteration were randomized while no patients with a tumor *FANCL* or *RAD51C* alteration were randomized. Despite PROfound’s large size (4425 patients screened, 387 patients randomized), only 3 of 15 genes included in cohorts A or B were altered in more than 20 patients (*BRCA2*, *ATM*, and *CDK12*). Therefore, given the lack of statistical power to determine the relative efficacy of PARP inhibition at the gene level for all but the most commonly altered genes, alternative approaches are needed to define the impact of specific genes on PARP inhibitor sensitivity.

Functional studies that directly test the impact of specific gene alterations on tumor biology and therapy response in prostate cancer preclinical models have the potential to complement data from mCRPC clinical trials^13,15,17^. However, prostate cancer preclinical studies are challenging due to a paucity of biologically relevant preclinical models as well as difficulty in creating viable models that harbor relevant DNA repair gene alterations. Here, using a series of prostate cancer cell lines, we show that *PALB2* or *BARD1* loss is sufficient to induce an HR-deficient phenotype and sensitize prostate cancer cells to PARP inhibition. Given that neither *PALB2* nor *BARD1* were altered at a sufficient frequency in the PROfound or TRITON2 trials to reliably determine the impact of each gene on PARP inhibitor sensitivity, these functional data fill an important gap by providing direct evidence that loss of *PALB2* or *BARD1* is sufficient to confer an HR-deficient phenotype in prostate cancer cells and suggest that alterations in *PALB2* or *BARD1* may be sufficient to drive PARP inhibitor sensitivity in mCRPC.

HR is a complex and highly regulated DNA repair pathway that involves numerous proteins that act at different steps of the repair process^33^. Some proteins such as BRCA1/2, PALB2, and BARD1 have a direct role at the DNA break whereas other such as ATM and Chk2 are involved in signal transduction and in coordinating repair activities with the cell cycle. Given the complexity of HR repair, it is not surprising that alterations in different genes in the HR pathway differentially impact HR activity and PARP inhibitor sensitivity in a context-dependent manner. Further contributing to the challenge of predicting PARP inhibitor sensitivity, different alterations in the same gene may confer dramatically different phenotypes. In this study we chose to model gene loss; however, many other types of HR gene alterations occur in mCRPC, including nonsense or frameshift mutations that are likely to result in loss of protein function as well as missense mutations that can vary widely in impact. Additional studies should focus on defining the effect of specific mutant DDR gene alleles on cellular properties and PARP inhibitor sensitivity. Furthermore, an HR-deficient phenotype and PARP inhibitor sensitivity are most likely to occur when both alleles are altered, either via biallelic loss/mutation or by mutation of one allele accompanied by loss of heterozygosity (LOH) of the second allele^29^.

Improving the ability to reliably predict the therapeutic implications of specific alterations in specific genes will require integrating data from clinical, genomic, and functional studies. In mCRPC, these efforts will become increasingly important as the number of treatment options continues to expand. Predictive biomarkers of PARP inhibitor response are needed so that PARP inhibitors can be directed to the subset of patients who are most likely to respond while patients less likely to respond to PARP inhibitor monotherapy can be prioritized for other approaches including PARP inhibitor combinations or other alternative targeted approaches.

## Methods

### Cell lines and reagents

Human prostate cancer lines DU145, 22Rv1, and LNCaP were purchased from ATCC and maintained in phenol red free RPMI 1640 (Gibco) supplemented with 10% heat inactivated FBS (Sigma). The U2OS DR-GFP HR reporter cell line was a gift from Dr. Alan D’Andrea (Dana-Farber Cancer Institute) and was maintained in DMEM (Gibco) supplemented with 10% FBS and 1% penicillin-streptomycin (Gibco). All cell lines were grown at 37 °C in a 5% CO_2_ incubator.

### HRD score calculations

Germline and somatic sequencing data for TCGA prostate cancer (PRAD) cohort were downloaded from https://portal.gdc.cancer.gov/projects/TCGA-PRAD and the variants were annotated using InterVar^34^. Allele-specific copy-number profiles were extracted using Sequenza^35^ and the whole exome sequencing-based HRD scores were determined using the scarHRD R package^36^.

### siRNA-mediated gene depletion

Two individual siRNAs targeting *PALB2* or *BARD1* were purchased from Integrated DNA Technologies and independently validated. See Supplementary Table 1 for siRNA sequences. A non-targeting (negative control) siRNA and an siRNA targeting *BRCA2* were purchased from Qiagen. Cells were seeded at 40% confluency in 6-well plates and transfected with 20 nM siRNA using Lipofectamine 3000 (Invitrogen) and Opti-MEM (Gibco). Transfected cells were incubated for 48 h and then re-plated for the desired assay.

### Immunoblotting

Gene depletion by siRNA was confirmed by immunoblotting. Briefly, cells were lysed with Pierce RIPA Buffer (Thermo Scientific) supplemented with phosphatase inhibitor (PhosSTOP, Roche), protease inhibitor (Roche), and 1 mM PMSF (Cell Signaling). Protein extracts were resolved on 4–12% NuPAGE Bis-Tris gradient gels (Invitrogen) and transferred to a 0.45-micron Nitrocellulose membrane (Thermo Scientific). Membranes were blocked with 5% milk in TBST and probed with primary antibody overnight at 4 °C. Primary antibodies were anti-PALB2 (1:1000, Bethyl Laboratories, clone A301-246A) and anti-BARD1 (1:1000, Santa Cruz, clone sc-74559). The following day, the membranes were washed and probed with secondary goat-anti-rabbit (IRDye 680RD; 1:10,000) and imaged using an Odyssey device (Li-Cor Biosciences).

### Immunofluorescence assays

Transfected cells were seeded onto glass coverslips and irradiated with 2 or 10 Gy using a cell irradiator (Rad Source). Cells were allowed to recover for 1–4 h, and then pre-extracted (20 mM HEPES pH 7.6, 50 mM NaCl, 3 mM MgCl_2_, 300 mM sucrose, 0.5% Tritonx-100) for 5 min on ice. Cells were gently washed once with PBS, fixed with formalin for 30 min, washed twice with PBS, and then blocked (1% Goat Serum, 100 mM Glycine in PBS, 0.2% TritonX-100) for 1 h at room temperature. Primary antibodies anti-RAD51 (1:500, Santa Cruz, sc-398587, clone F-11) and anti-yH2AX (1:500, Millipore 05-636, clone JBW301) were diluted in blocking solution and incubated overnight at 4 °C. Cells were washed with PBS 3X for 15 min and then incubated with secondary antibody (AlexaFluor594 goat-anti-mouse, 1:2000, A11032 or AlexaFluor488 goat-anti-mouse, 1:2000, A11001) diluted in blocking solution for 1 h at room temperature. Coverslips were washed with PBS 3X for 15 min and then mounted onto glass slides using Fluoro-Gel II (Electron Microscopy Sciences, #17985-50). Foci were quantified by setting a threshold of 10 discrete foci per cell. The number of cells per well that had ≥10 RAD51 foci were counted in triplicate and a percent was calculated based on DAPI positive nuclei.

### DR-GFP assay

U2OS or DU145 cells stably expressing the DR-GFP expression cassette (U251-DR-GFP) were seeded in 6-well plates and transfected with siRNA. After 24 h, cells were infected with I-SceI adenovirus and FACS was performed 36 h following infection. FACS was performed using a CytoFLEX flow cytometer (Beckman-Coulter) and the percentage of viable GFP-expressing cells was calculated.

### Drug sensitivity assays

Forty-eight hours following siRNA transfection, cells were seeded into 96-well plates (5000 cells/well) in triplicates and treated with the PARP inhibitor olaparib or rucaparib (Selleck Chemicals). Cell viability was measured at 72 h using CellTiterGlo (Promega). For clonogenic survival assays, transfected cells were seeded into 6-well plates (1000 cells/well) in triplicate and treated with olaparib for 10–14 days. Plates were fixed with formalin (Sigma) for 30 min at room temperature and then stained with crystal violet solution.

### Statistical analyses

All cell line experiments were performed in triplicate and data is plotted as the mean ± standard deviation of multiple independent experiments. Prism 7 (GraphPad) was used to display and quantify data. For the cell line experiments, student’s *t* tests were used to compare difference between means and *p* values < 0.05 were considered to be statistically significant.

### Ethics approval

All work was performed at the Dana-Farber Cancer Institute and the Danish Cancer Society Research Center and these institutions gave ethical approval for the study.

### Reporting summary

Further information on research design is available in the Nature Research Reporting Summary linked to this article.

## Supplementary information

Supplementary Material

PALB2 and BARD1 mutant cases from TCGA PRAD cohort

REPORTING SUMMARY

## Data Availability

No custom code or scripts were created to perform the data analyses in this study. Graphs were made using GraphPad Prism (v9.3.1) or R 3.6.3 with the ggplot2 package.

## Code availability

Code used to generate figures during the current study are available from the corresponding author upon request.
