# Supplementary material for: PALB2 or BARD1 loss confers homologous recombination deficiency and PARP inhibitor sensitivity in prostate cancer
Source: NPJ Precis Oncol. 2022 Jun 29;6:49. doi: 10.1038/s41698-022-00291-7 (PMC9242979; doi:10.1038/s41698-022-00291-7)
Supplement: Supplementary file 1 — Supplementary Material [file 41698_2022_291_MOESM1_ESM.pdf]

Supplementary Figure 1

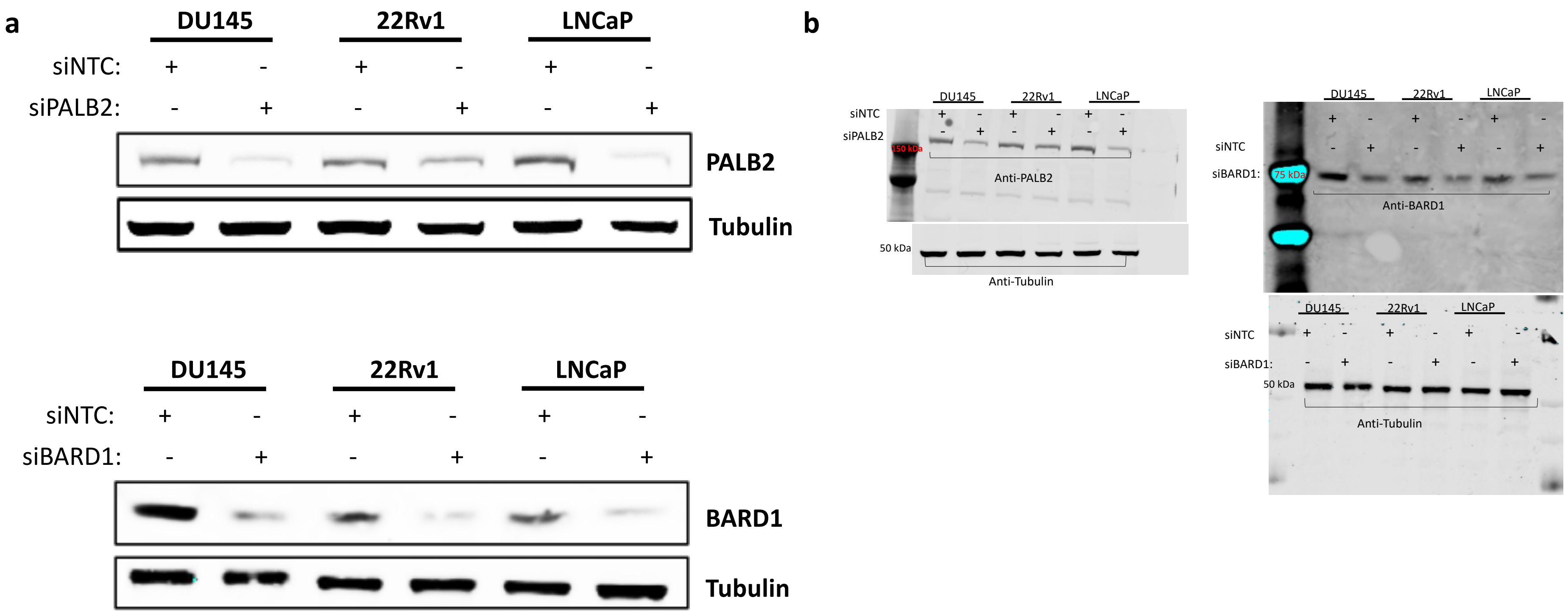

Supplementary Figure 1: Immunoblot showing siRNA-mediated depletion of PALB2 or BARD1 in prostate cancer cell lines DU145, 22Rv1, and LNCaP. **a.** Cropped blot images with optimized exposure and accompanying loading controls. **b.** Uncropped images of same blots with adjacent molecular weight markers. All blots were derived from the same experiment and were processed in parallel.

Supplementary Figure 2

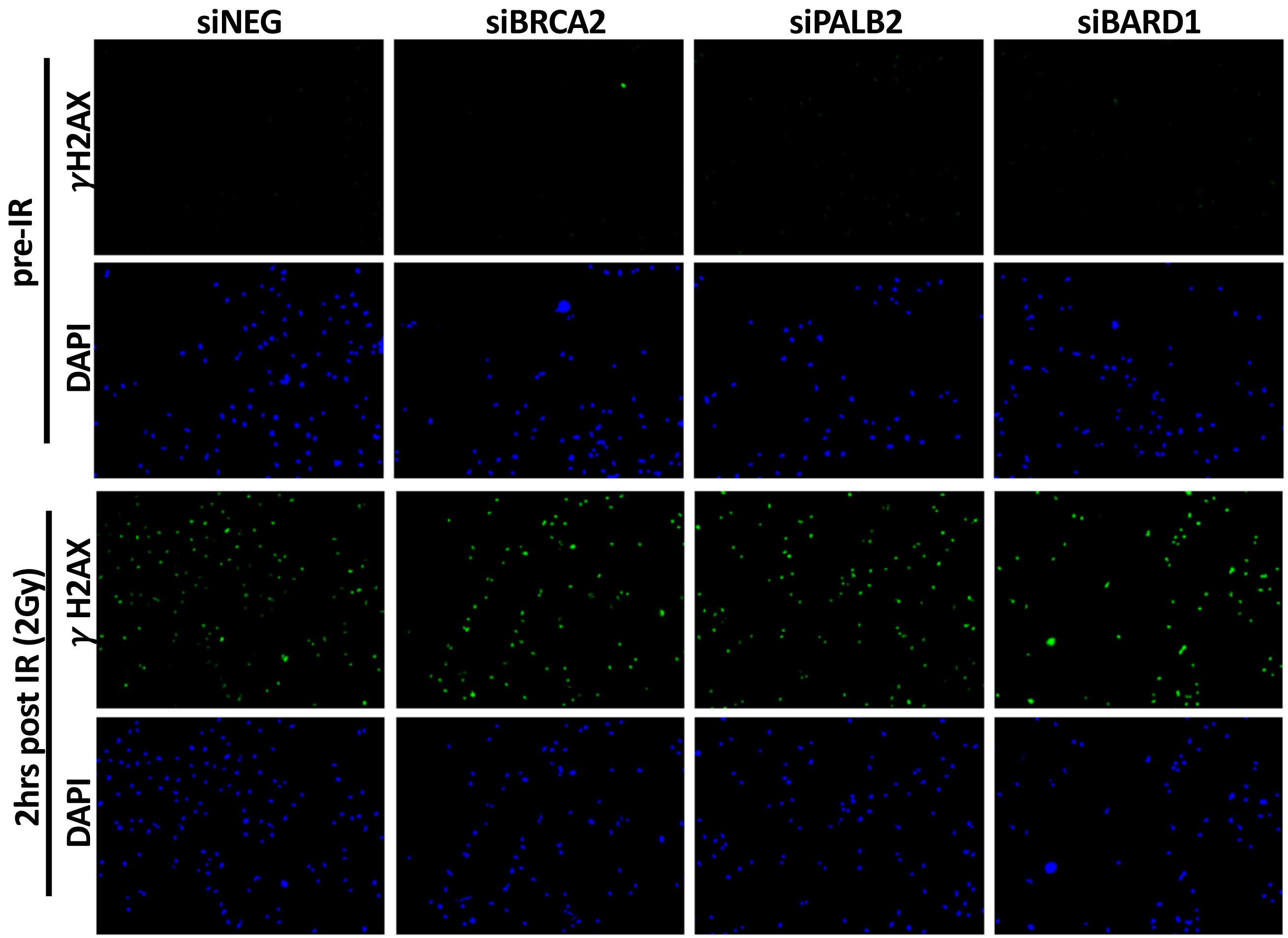

Supplementary Figure 2: Low-power (20X) images of IR-induced γH2AX foci in DU145 cells. None of the cells have γH2AX foci prior to IR. Following IR, all cells transfected with non-targeting siRNA (siNEG) or siRNA targeting BRCA2, PALB2, or BARD1 form γH2AX foci.

Supplementary Figure 3

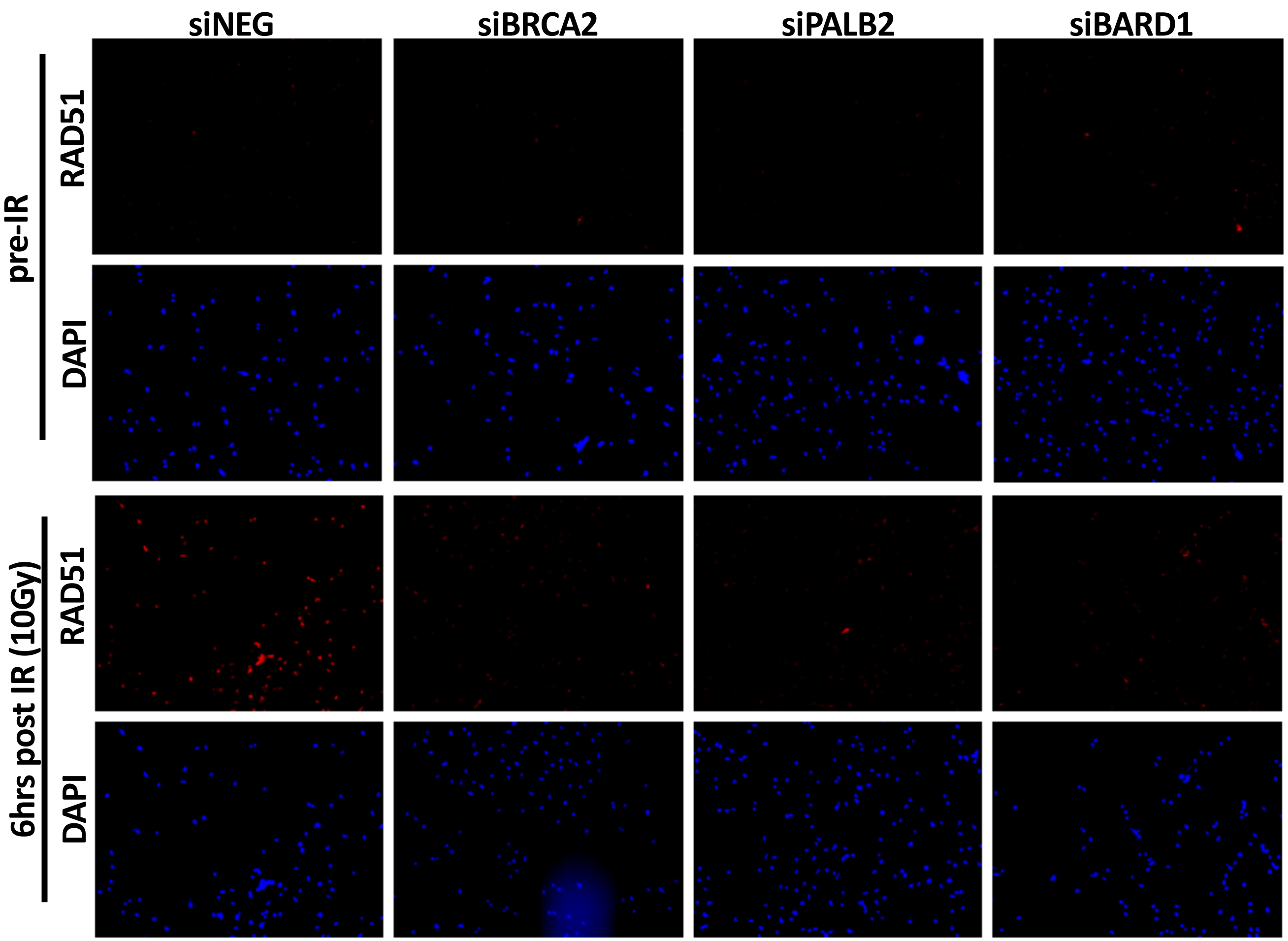

Supplementary Figure 3: Low-power (20X) images of IR-induced Rad51 foci in DU145 cells. None of the cells have Rad51 foci prior to IR. Following IR, cells transfected with non-targeting siRNA (siNEG) form Rad51 foci whereas cells transected with siRNA targeting BRCA2, PALB2, or BARD1 fail to form Rad51 foci.

Supplementary Figure 4

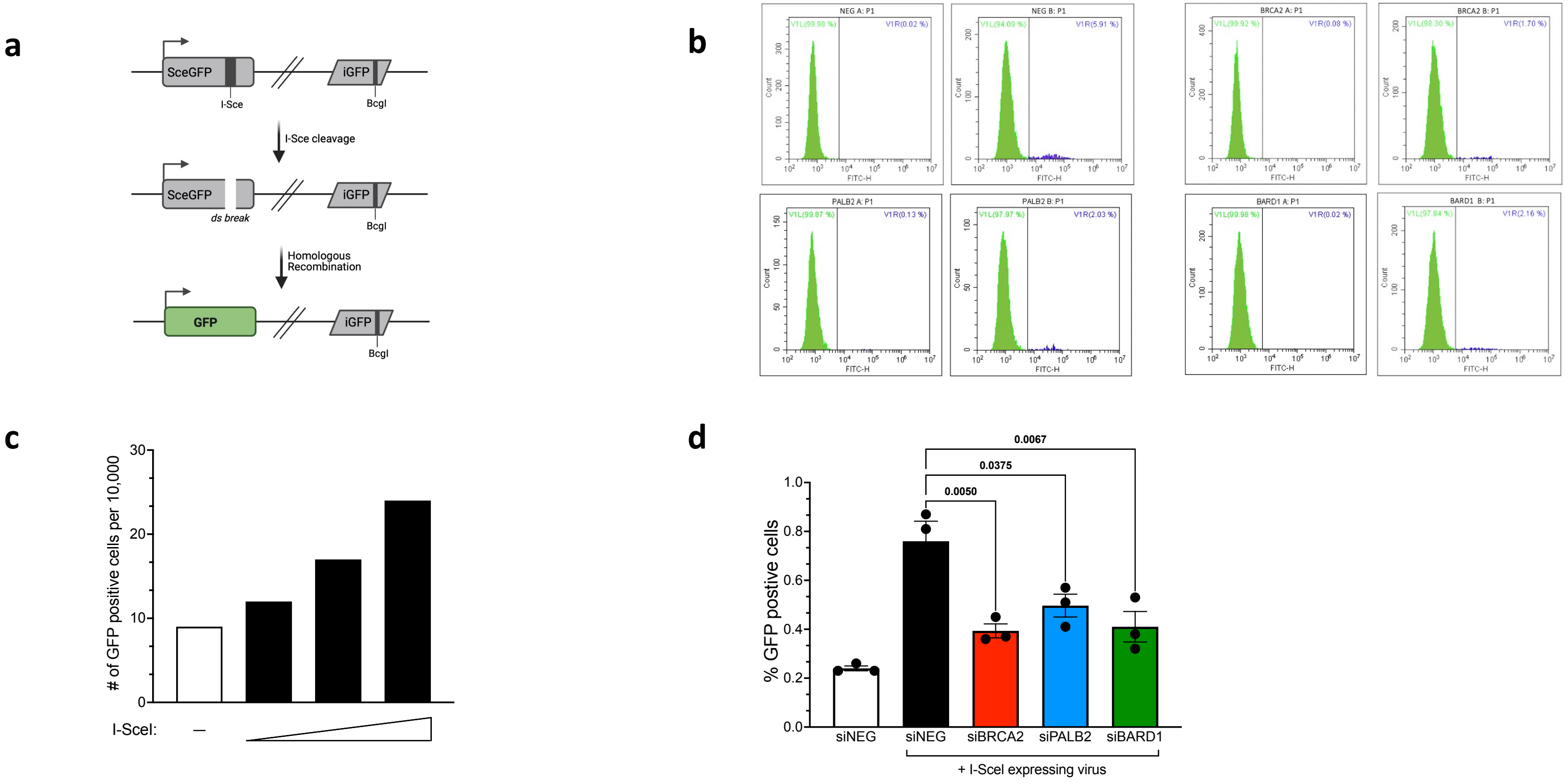

Supplementary Figure 4: DR-GFP assay. **a.** Schematic overview of the DR-GFP assay. **b**. The single parameter FACS gating strategy used for quantification of GFP-positive and GFP-negative cells in the DR-GFP assay is denoted by the horizontal line in each panel. **c**. The percentage of GFP-positive DU145 prostate cancer cell increases as the amount of I-SceI adenovirus used to infect the cells was increased. **d**. HR-mediated repair of an induced double-strand break is significantly lower in PALB2- and BARD1-depleted DU145 cells compared to mock-depleted cells (siNEG). BRCA2-depleted cells are included as a control for HR deficiency. GFP, green fluorescent protein. HR, homologous recombination.

Supplementary Figure 5

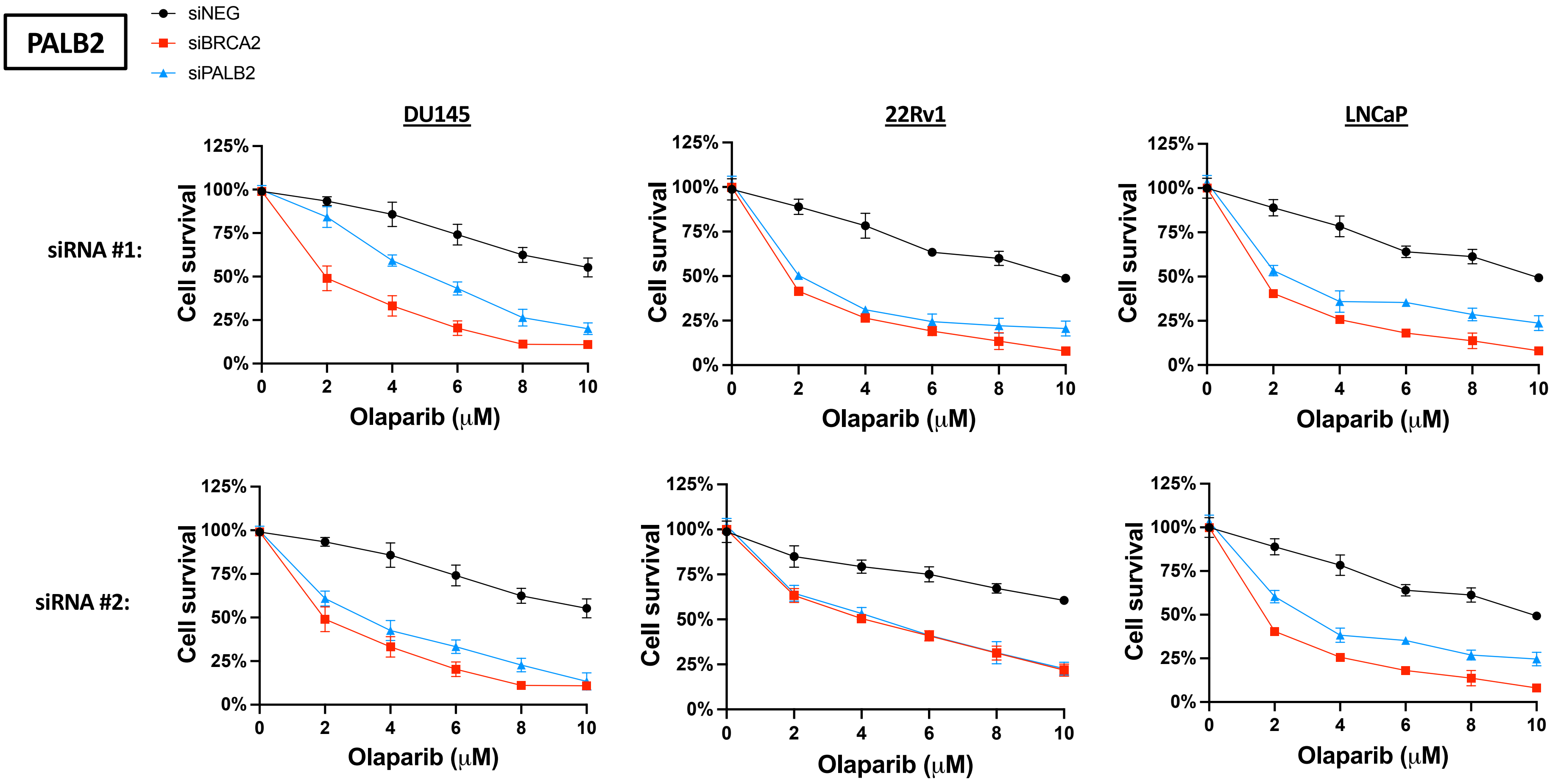

Supplementary Figure 5: PARP inhibitor sensitivity of prostate cancer cell lines following depletion of PALB2 with two independent siRNAs. Loss of PALB2 increases sensitivity to the PARP inhibitor olaparib to a similar extent as BRCA2 loss. Error bars represent standard deviation of values from experiments performed in triplicate.

Supplementary Figure 6

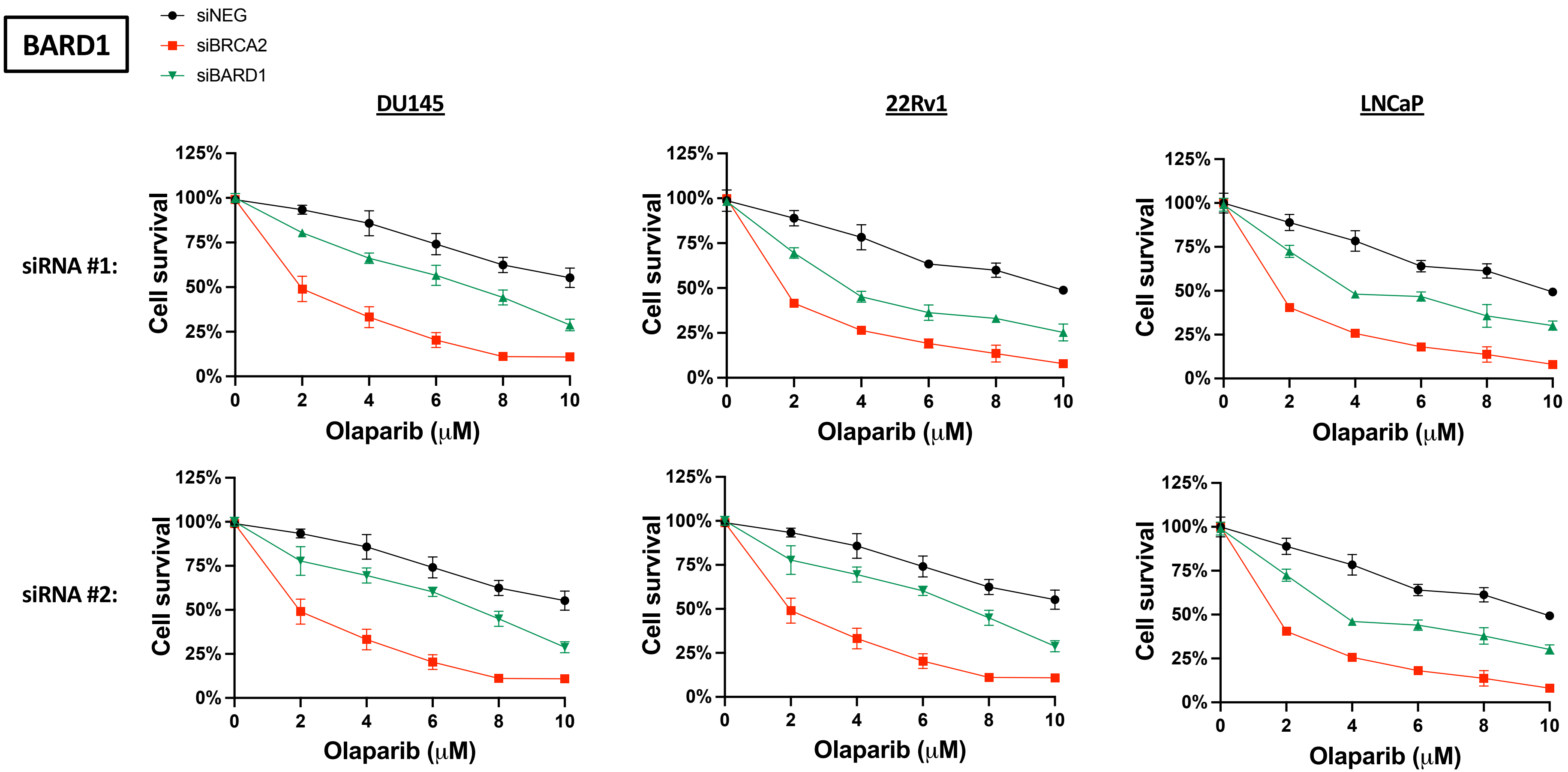

Supplementary Figure 6: PARP inhibitor sensitivity of prostate cancer cell lines following depletion of BARD1 with two independent siRNAs. The impact of BARD1 loss on sensitivity to thePARP inhibitor olaparib is less than the impact of BRCA2 loss. Error bars represent standard deviation of values from experiments performed in triplicate.

Supplementary Figure 7

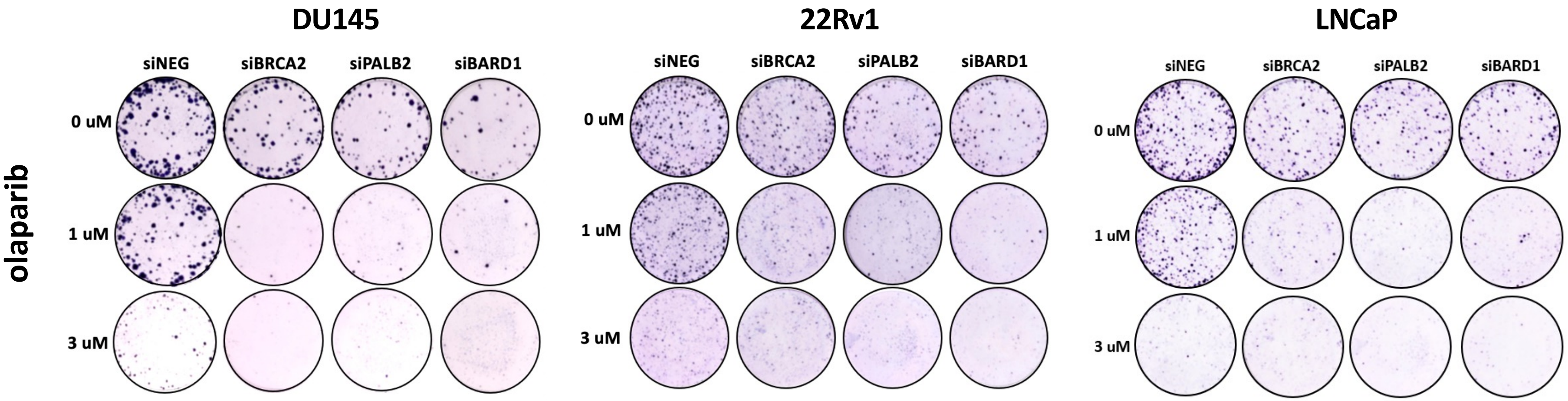

Supplementary Figure 7: Representative images of clonogenic survival following PARP inhibitor treatment in prostate cancer cell lines with depletion of BRCA2, PALB2, or BARD1. Depletion of BRCA2, PALB2, or BARD1 results in a significant increase in PARP inhibitor sensitivity compared to mock depletion (siNEG).

Supplementary Figure 8

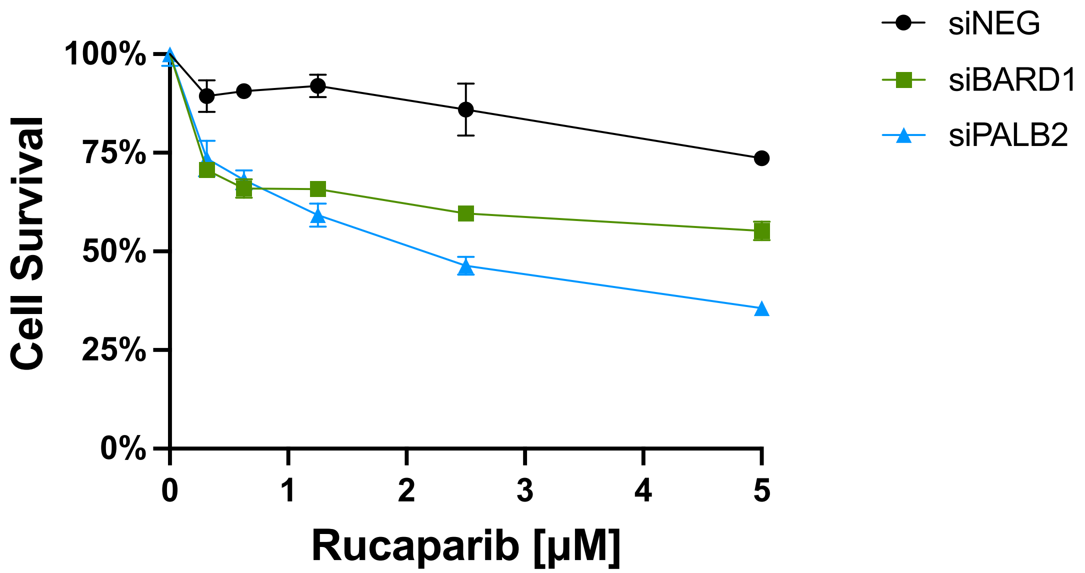

Supplementary Figure 8: Rucaparib sensitivity of DU145 prostate cancer cell line following mock depletion or depletion of PALB2 or BARD1. Error bars represent standard deviation of values from experiments performed in triplicate.
